# Genomics of Obsessive-Compulsive Disorder—Toward Personalized Medicine in the Era of Big Data

**DOI:** 10.3389/fped.2021.685660

**Published:** 2021-10-20

**Authors:** Natalia Szejko, Anna Dunalska, Adam Lombroso, Joseph F. McGuire, John Piacentini

**Affiliations:** ^1^Department of Neurology, Yale School of Medicine, New Haven, CT, United States; ^2^Department of Neurology, Medical University of Warsaw, Warsaw, Poland; ^3^Department of Bioethics, Medical University of Warsaw, Warsaw, Poland; ^4^Child Study Center, Yale School of Medicine, New Haven, CT, United States; ^5^Department of Psychiatry and Behavioral Sciences, Johns Hopkins University School of Medicine, Baltimore, MS, United States; ^6^Semel Institute of Neuroscience and Human Behavior, University of California, Los Angeles, Los Angeles, CA, United States

**Keywords:** genomics, genetics, obsessive-compulsive disorder, Big Data, genome-wide association study

## Abstract

Pathogenesis of obsessive-compulsive disorder (OCD) mainly involves dysregulation of serotonergic neurotransmission, but a number of other factors are involved. Genetic underprints of OCD fall under the category of “common disease common variant hypothesis,” that suggests that if a disease that is heritable is common in the population (a prevalence >1–5%), then the genetic contributors—specific variations in the genetic code—will also be common in the population. Therefore, the genetic contribution in OCD is believed to come from multiple genes simultaneously and it is considered a polygenic disorder. Genomics offers a number of advanced tools to determine causal relationship between the exposure and the outcome of interest. Particularly, methods such as polygenic risk score (PRS) or Mendelian Randomization (MR) enable investigation of new pathways involved in OCD pathogenesis. This premise is also facilitated by the existence of publicly available databases that include vast study samples. Examples include population-based studies such as UK Biobank, China Kadoorie Biobank, Qatar Biobank, *All of US* Program sponsored by National Institute of Health or Generations launched by Yale University, as well as disease-specific databases, that include patients with OCD and co-existing pathologies, with the following examples: Psychiatric Genomics Consortium (PGC), ENIGMA OCD, The International OCD Foundation Genetics Collaborative (IOCDF-GC) or OCD Collaborative Genetic Association Study. The aim of this review is to present a comprehensive overview of the available Big Data resources for the study of OCD pathogenesis in the context of genomics and demonstrate that OCD should be considered a disorder which requires the approaches offered by personalized medicine.

## Introduction

Obsessive-compulsive disorder (OCD) is a common disorder with a population prevalence of 2–3% (1). Moreover, up to 13% of adults experience some kind of obsessive-compulsive behaviors (OCB) during their lifetime (1). Obsessive-compulsive disorder has chronic course with child onset in 50–70% of cases, typically associated with significant impairment and comorbidity. The spectrum of obsessive-compulsive (OC) symptoms varies from non-bothersome intrusive thoughts and compulsive behaviors (OCB) to full-blown OCD. Irrespective of where someone falls along the spectrum, a primary contributing factor to this OC spectrum is dysregulation of neurotransmission, mainly the serotonergic system (2–4). Nevertheless, other factors have also been found to influence the occurrence of OCD (5, 6), in particular, brain injury (7), toxicity (8), infection, and autoimmunity, especially in context of pediatric autoimmune neuropsychiatric disorders associated with streptococcal infections (PANDAS) and pediatric acute-onset neuropsychiatric syndrome (PANS) (9, 10) and genetics (11). A number of candidate genes have been proven to play a role in pathogenesis of OCD, mainly related to serotonergic, glutamatergic, and dopaminergic pathways (12), but recent studies have demonstrated that OCD occurrence is multifactorial and probably is a consequence of gene/environment interactions (13, 14). Moreover, although it has been assumed that serotonergic mechanisms are important for OCD, this still needs to be empirically proven by unbiased genome wide association studies (GWAS).

A surge of new genetic technologies, such as GWAS, has enabled much more precise analysis of the genetic underprints of diseases. Genome wide association studies is an observational study of a genome-wide set of genetic variants in different individuals to determine if any variant is associated with a trait of interest (15). Genome wide association studies typically focuses on associations between single nucleotide polymorphisms (SNPs) and the outcome of interest. Consequently, SNPs are a substitution of a single nucleotide at a specific position in the genome that is present in a sufficiently large fraction of the population (16). Furthermore, blooming of advanced statistical and mathematical methods facilitate even more precise discoveries in the area of genetics. In particular, a method called Mendelian Randomization (MR) (17) enables establishment of casual relationship between genetically determined risk factors and the phenotype of interest, in this case, OCD. Moreover, by using external GWAS results with a list of SNPs that have reached genome wide significance and genotyping a particular individual it is possible to estimate genetic risk related to an outcome of interest. This risk is described as a numeric value denominated polygenic risk score (PRS) (18). Genome wide association studies, MR, PRS, and SNPs are the basic terms used in population genetics, the field of genetics that derives from epidemiological studies. Creation of research consortia that enable agglomeration of more and more data is a key mechanism that facilitates research in this area. At the same time, scientists are facing the problem of too many data that, due to its huge volume, are denominated Big Data. The concept of Big Data falls under the umbrella of the acronym “3 V” model: volume, velocity and variety. Rapid development of all aforementioned areas also has repercussions on the discoveries regarding pathophysiology of OCD.

The aim of this review is to present an overview of the available consortium and Big Data resources gathering OCD-related data and how these, and other resources are used to unravel genetics of OCD. Finally, we would like to demonstrate how tools provided by population genetics and genomics enable personalized diagnosis and treatment of OCD.

## Most Widely Used Tools From the Field of Population Genetics

Population genetics offers powerful possibilities to overcome limitations of observational studies and demonstrate causal inference. As mutations are randomly distributed during meiosis, mutation-disease associations are not influenced by confounding post-natal factors. Population genetics uses a number of techniques and analytical methods that enable determination of casual link between the exposure and the outcome. Candidate gene studies were the main method to test associations between genes and diseases before the development of more advanced genotyping technologies (19). This approach is hypothesis-driven and is supported by the specific supposition in which specific biological pathway is related to the final phenotype. Genome wide association studies, on the other hand, are not precluded by the hypothesis-driven approach as they enable investigation of millions of SNPs across the genome for association with a particular disease. In this case, it is standard to use an adjusted threshold for statistical significance of *p* < 5 × 10e^−8^ to account for the approximately 1 million independent loci found across the human genome. Genome wide association studies technique enabled creation of another important statistical tool, PRS. This allows creation of scores that summarize the load of mutations related to a specific trait. Polygenic risk score is a sum of risk alleles for a given person, which is often more powerful predictor of disease occurrence than an individual SNP. Finally, MR is a statistical method aimed at determining and quantifying causal relationships between genetically-determined exposures and outcomes of interest (20). Importantly, in contrast to randomized clinical trials, the most frequently used tool to evaluate causality, MR can be performed using already available open-access data from different sources, allowing the evaluation of larger numbers of possible mechanisms and accelerating the speed of the translational cycle.

## Big Data Resources and Consortia in Population Genetics

The growing amount of data and information in field of medicine is offering a number of new opportunities, but is also a major challenge, both in terms of data storage as well as analysis. Expansive development and use of new technologies, adopted from the fields of bioinformatics, statistics, and mathematics, help scientists analyze these data in a proper manner, and interpret the results. Moreover, the new research philosophy, based on the construction of large international and intercontinental consortia, permits researchers to overcome previous methodological limitations, mainly related to small sample size. Importantly, in accordance with the inclusive nature of research, more and more data are publicly available. As vast majority of common diseases, such as cardiovascular conditions or mental disorders, are multifactorial; they are result of the complex interactions of genes and environment (21). These disorders also fall under the category of the common disease, common variant hypothesis (21), which argues that genetic variations with appreciable frequency in the population at large, but with relatively low penetrance, are the major contributors to genetic susceptibility to common diseases. This means that large samples are required to study associations between these exposures and disease, and to identify targets for treatment and prevention.

In recent years, several population-based initiatives were initiated in order to collect demographic, epidemiological, clinical, neuroimaging, biomarkers, and genetic data. The most relevant examples include such projects as UK Biobank, *All of US* Program sponsored by National Institute of Health, Generations by the Yale University, China Kadoorie Biobank or Qatar Biobank. All of these databases contain data about psychiatric health and symptoms and could be used to investigate a number of questions related to OCD pathophysiology. The most robust and well-described study is UK Biobank (www.ukbiobank.ac.uk). UK Biobank is a population-based cohort and biobank investigating contributions of genetic predisposition and environmental exposure to the development of disease. The study was initiated in 2006, included over half a million people aged 40–69 years at onset, and proposed long-term follow-ups. Recruitment was finalized in 2010 and the resource is constantly growing. In 2017, genotyping of all participants was completed (22), in 2019 a wide range of biomarkers was released, and multimodal neuroimaging for almost 100,000 participants is gradually being published (23). The design of the UK Biobank study facilitates exploration of an extensive range of diverse risk factors and outcomes and provides tools to detect small effects in a large study sample. Importantly, UK Biobank also provides baseline and follow-up data on mental disorders, including OCD (24).

Several population-based studies were launched in the US. National Institute of Health initiated *All Of US* (https://allofus.nih.gov/) program which aims to enroll 1 million adults across the US. This study was initiated in 2015 under the government of Barack Obama and is a reflection of the efforts aiming to popularize precision medicine. As indicated in Carrosco-Ramiro et al. (25), precision or personalized medicine derives from the advances in genetic/genomic techniques and the completion of the Human Genome Project (HGP). Precision medicine incorporates information from genome sequencing and clinical data which enables therapy adjustment according to patient's own genome and environmental factors. Importantly, precision medicine is executed in line with the following premises: predictive, preventive, personalized, and participatory (P4). *All Of US* is destinated to facilitate the implementation of the P4 principles on a population level. Therefore, participation in this project is voluntary, independent of sex, gender, or ethnicity, and reflects the rich diversity of the US. The study is totally transparent as each participant receives individual results, including their genetic data. Participants provide clinical data and can provide additional access to their electronic health records (EHR) which include all their information about health problems as well as any medications they take. Blood and urine samples, as well as physical measurements, including those gathered by wearable devices, are also collected. In the future this program is planned to facilitate execution of clinical trials. In addition, blood samples are genotyped. By June 2020, enrollment reached approximately 350,000 individuals. Eighty percent of those people are from groups that have been traditionally underrepresented in biomedical research making *All of US* the first study focused on diversity. The Million Veteran Program (26) (https://www.mvp.va.gov/) is another innovative study sponsored by the Department of Veterans Affairs Office of Research and Development. So far, it has been possible to enroll 825,000 individuals. Similar to previous cohorts, demographic and clinical data, as well as biological samples were collected. Importantly, genotyping has already been conducted and enables testing of many hypotheses related to psychiatric diseases (27). Yale's Generations project (https://medicine.yale.edu/ycci/trial/6326/) was launched in 2019 and is targeted to be another precision medicine cohort. It will gather genetic and clinical data from at least 100,000 participants, including pediatric participants. DNA patterns will be linked to EHR.

Another important resource is China Kadoorie Biobank (https://www.ckbiobank.org/). It enabled the acquisition of genomic and clinical data on chronic diseases in half a million Chinese participants (28). The baseline data collection was carried out in years 2004–08 and included biological samples, along with demographic and clinical data. Participants were aged 30–79 years old. A select subset of participants is also retested every few years. Similar to UK Biobank, data regarding mental disorders are also available (29–31). Analogous projects were also launched in Japan (https://biobankjp.org/english/index.html) and Qatar (https://www.qatarbiobank.org.qa/home).

All in all, Big Data resources enable quick and unlimited access to previously restricted resources as researchers from all over the world can solicit permit to work on the data of interest. This can lead to democratization of science. Moreover, thanks to these resources, it is possible to investigate both common and rare diseases. Finally, the sample size is large enough to achieve previously unthinkable statistical power. The majority of biobanks offer not only information at baseline, but also follow-up, which enable high-quality longitudinal analysis. Finally, biobanks, in contrast to cohort studies, gather complex clinical, neuroimaging, and genetic data, not only about one restricted disease, but whole variety of phenotypes or even enroll mainly healthy individuals. Good example is previously described UK Biobank aiming to enroll any middle aged individual or Health and Retirement Study at University of Michigan, study investigating the dynamics of aging. As a result, information provided by Big Data resources is more approximated to the distribution of phenotypes and risk factors in the population.

## Big Data Resources and Consortia Related to Obsessive-Compulsive Disorder

Apart from population-based Biobanks, a number of cohorts related to OCD and/or mental health could be used to investigate OCD phenotype variability. However, the results obtained from diverse studies differ due to differences in the sample collection and diverse description of clinical phenotype. For example, ENIGMA OCD protocol includes only participants with available neuroimaging data while the majority of other cohorts did not include this criterion. Therefore, these data have to be interpreted with caution, taking together all the limitations mentioned.

Psychiatric Genomics Consortium (PGC) (https://www.med.unc.edu/pgc/) (32–34) incorporates more than 800 scientists worldwide coming from more than 150 institutions and 40 countries. One of the nine disorders working groups is dedicated to OCD and Gilles de la Tourette syndrome (GTS) and is headed by primary investigators in genetics of these disorders, Jeremiah Scharf and Manuel Mattheisen (13, 35). Participation in PGC is inclusive as anyone willing to contribute with samples can take part in the entire data analysis. The majority of data are available upon request.

The International OCD Foundation Genetics Collaborative (IOCDF-GC) (https://iocdf.org/programs/genetics/) (13) is a group of genetics investigators from North America, South America, Europe and Africa who collect data from OCD patients for genetic analysis, including GWAS (detailed results presented in section Genomics of Obsessive-Compulsive Disorder).

OCD Collaborative Genetic Association Study (OCGAS) (36) is a six-site, collaborative, genetic linkage study of OCD. Specimens and blinded clinical data are made available through the National Institute of Mental Health repository. In this project, clinical data and blood specimens were collected from 238 families containing 299 OCD-affected sibling pairs and their parents, and additional affected relative pairs, for a GWAS (detailed results presented in section Genomics of Obsessive-Compulsive Disorder).

ENIGMA OCD (http://enigma.ini.usc.edu/ongoing/enigma-ocd-working-group/) (37– 40) currently consists of 47 samples from 34 institutes in 15 countries on 5 continents, with a total sample of 2,323 OCD patients and 2,325 healthy controls. The main aim of this consortium is to collectively analyze brain imaging, clinical, and genetic data. Initially formed to detect genetic influences on brain measures, ENIGMA has grown to over 30 working groups studying 12 major brain diseases and comparing brain data. The total number of enrolled subjects so far is of 2,323 OCD patients and 2,325 healthy controls. Although vast majority of studies focused on different modalities of neuroimaging investigating subcortical volume (41), cortical thickness (42), structural connectivity (38), or brain lateralization (37), there are reports about correlation between genomic and neuroimaging data (43, 44). Recent efforts have focused on using modern technologies, in particular machine learning (39).

Table 1 summarizes Big Data resources in population genetics and related to OCD, in particular.

**Table 1:** Consortia and Big Data initiatives related to obsessive-compulsive disorder.

**Name of the consortium/database**	**Website**	N of participants^**^	**Age of participants**	**Ethnicity of participants**	**Country of inclusion**
UKB	www.ukbiobank.ac.uk	500,000	40–69	94.6% of participants are of white ethnicity	UK
*All Of US*	https://allofus.nih.gov	1 million	>18	Diverse	US
The Million Veteran Program	https://www.mvp.va.gov/	825,000	>18	Diverse	US
Yale's Generations	https://medicine.yale.edu/ycci/programsprojects/generations/	100,000	No age limits	Diverse	US
China Kadoorie Biobank	https://www.ckbiobank.org/site/	510,000	>18	Asian	China
Biobank Japan	https://biobankjp.org/english/index.html	200,000	>18	Asian	Japan
Qatar Biobank	https://www.qatarbiobank.org.qa/home	60,000	>18	Arabic	Qatar
Psychiatric Genomics Consortium	https://www.med.unc.edu/pgc/	25,000	No age limits	Diverse	International
The International OCD Foundation Genetics Collaborative	https://iocdf.org/programs/genetics/	1,429 cases; 5,089 controls	No age limits	European	International
OCD Collaborative Genetic Association Study	https://www.ncbi.nlm.nih.gov/pmc/articles/PMC2555990/	344 cases and 1,033 controls	No age limits	European	International

*UKB, UK Biobank*.

** *included or targeted*.

## Genomics of Obsessive-Compulsive Disorder

As mentioned in the introduction, research on complex diseases has been revolutionized by GWAS, which enables the simultaneous analysis of SNPs and the search for statistical relationships between them. This type of analysis, based on the achievements of modern genomic technologies, goes beyond the possibilities of candidate gene association studies and creates the possibility to discover genetic risk factors for diseases without the need to select specific genes and formulate a priori hypotheses (58). The main difference between genomics and genetics is that genetics focuses on functioning and composition of the single gene whereas genomics addresses all genes and their relationships to each other in order to identify their combined influence on the growth and development of the organism (59). In the following sections we discuss studies tackling the topic of genomics of OCD (Table 2). Findings provided by studies targeting the genomics of OCD are of great importance since only these studies could help to unravel complex genetic architecture of OCD. As a consequence, they can help to find pathophysiological pathways involved in the occurrence of OCD and plan treatment, especially in the context of personalized medicine. Nevertheless, results of these studies are often contradictory as studies included different sample size and included participants with diverse phenotype. This is the case for other GWAS examing genetic background of heterogenous traits, such as height (60), diabetes (61), and schizophrenia (62).

**Table 2:** The most important studies investigating genomics of OCD.

**Study**	**Sample size**	**Population**	**Genes/SNPs identified**	**Pathway/pathology**
Stewart et al. (45)	1,465 cases, 5,557 ancestry-matched controls and 400 complete trios	European, South African and Ashkenazi Jewish	rs6131295, *BTBD3, DHRS11, ISM1, FAIM2, ADCY8, DLGAP1*	Cytoskeleton dynamics, ion channel modulation and protein degradation, glutamatergic neurotransmission, post-synaptic density of glutamatergic synapses
Mattheisen et al. (46)	5,061	European	rs4401971, *PTPRD, CDH9, IQCK, C16orf88, DLGAP1, GRIK2, NEUROD6, SV2A, GRIA4, SLC1A2*	Differentiation of glutamatergic and GABAergic synapses, early neurodevelopment
den Braber et al. (47)	6,931	NR	rs8100480, *MEF2BNB, RFXANK, MEF2BNB-MEF2B, MEF2B*	Immune system functions, muscle-specific genes' expression
Qin et al. (48)	804	NR	rs17162912, *DISP1*, rs9303380, rs12437601, rs16988159, rs723815, rs7676822, rs1911877, *GRIN2B, PCDH10, GPC6*	Glutamatergic and serotonergic neurotransmission
Umehara et al. (49)	96	Asian (Japanese)	CHN2	Calcium signaling
Guo et al. (50)	9,896	European	rs4785741, *MC1R, TUBB3, DDAH1, IMPA2, PTH2R*	Hair color, pigmentation, neurogenesis, CVDs, susceptibility to bipolar disorder, PTH
IOCDF-GC and OCGAS (13)	9,725	European	rs4733767, *CASC8/CASC11*, rs1030757, *GRID2*, rs12504244, *KIT, ASB13, RSPO4, DLGAP1, PTPRD, GRIK2, FAIM2, CDH20*	Glutamatergic neurotransmission
Khramtsova et al. (51)	9,870	European	*GRID2, GPR135*	Glutamatergic signaling system
Cross-Disorder Group of the Psychiatric Genomics Consortium (2019) (52)	727,126	European	109 pleiotropic loci	Neurodevelopment
Alemany-Navarro et al. (53)	399	European	*SETD3, CPE*	Zinc ion response and lipid metabolism, lipid metabolism, G protein-mediated processes, metabolic processes, and anion transport
Costas et al. (54)	813	European	*DNM3*	Endocytosis of synaptic vesicles
Smit et al. (55)	8,267	European	*KIT, GRID2, WDR7, ADCK1*	Emotional, reward processing, memory, fear-formation functions
Burton et al. (56)	5,018	European	rs7856850 (*PTPRD)*	Differentiation of neurons
Strom et al. (57)	390,290	European	PRS of neuroticism, bipolar disorder, anorexia nervosa, age at first birth, educational attainment, and insomnia	Neuroticism, bipolar disorder, anorexia nervosa, age at first birth, educational attainment, and insomnia

*Studies are listed in chronological order. SETD3, SET domain containing 3 gene; PTPRD, protein tyrosine phosphatase δ gene; CPE, carboxypeptidase E gene; DNM3, dynamin 3 gene; MEF2BNB, myocyte enhancer binding factor 2B gene; RFXANK, DNA-binding protein RFXANK gene; MC1R, melanocortin 1 receptor gene; TUBB3, tubulin beta 3 gene; DDAH1, dimethylarginine dimethylaminohydrolase 1 gene; IMPA2, inositol monophosphatase 2 gene; PTH2R, parathyroid hormone 2 receptor; CASC8/CASC11, cancer susceptibility 8 and cancer susceptibility 11 genes; GRID2, glutamate ionotropic receptor delta type subunit 2 gene; KIT, proto-oncogene, receptor tyrosine kinase gene; ASB13, ankyrin repeat and SOCS box containing 13 gene; RSPO4, R-spondin 4 gene; DLGAP1 gene, discs large homolog associated protein 1; FAIM2, fas apoptotic inhibitory molecule 2 gene; CDH20, cadherin 20 gene; GPR135, G protein-coupled receptor 135 gene; CDH9, cadherin 9 gene; IQCK, IQ motif containing K gene; NEUROD6, neuronal differentiation 6 gene; SV2A, synaptic vesicle glycoprotein 2A gene; GRIA4, glutamate ionotropic receptor AMPA type subunit 4 gene; SLC1A2, solute carrier family 1 member 2 gene; WDR7, WD repeat-containing protein 7 gene; ADCK1, AarF domain-containing protein kinase 1 gene; BTBD3, BTB domain-containing 3 gene; DHRS11, dehydrogenase/reductase 11 gene; ISM1, isthmin 1 gene; ADCY8, adenylate cyclase type 8 gene; DISP1, dispatched RND transporter family member 1 gene; GRIN2B, glutamate ionotropic receptor NMDA type subunit 2B gene; PCDH10, protocadherin 10 gene, GPC6, glypican 6 gene, CHN2, chimerin 2 gene; LRRC16A, leucine-rich repeat-containing 16A gene; PRS, polygenic risk score*.

## GWAS Findings in OCD

Important attempt to determine the genetic variation responsible for OCD was a study performed by Stewart et al. (45). To tackle this problem, IOCDF collected a set of individuals affected with OCD, diagnosed according to Diagnostic and Statistical Manual of Mental Disorders (DSM-IV) criteria, a subset of their parents, and unselected controls. Participants were then genotyped with Illumina SNP microarrays, which reduced the group to 1,465 cases, 5,557 ancestry-matched controls, and 400 parent–child trios. Study revealed a significant enrichment of methylation quantitative trait locus (QTLs) (*p* < 0.001) and frontal lobe expression quantitative trait loci (eQTLs) (*p* = 0.001) within the top-ranked SNPs (*p* < 0.01) in the combined trio-case-control sample, but no SNPs associated with OCD at a genome-wide significance level were recognized. The analysis including trios one SNP, rs6131295, located near the BTB domain-containing 3 (*BTBD3)* gene, reached genome wide statistical significance (*p* = 3.8 × 10^−8^), but in the combined trio-case-control meta-analysis this significance was not maintained. The abovementioned SNP is an eQTL for *BTBD3*, dehydrogenase/reductase 11 (*DHRS11)*, and isthmin 1 (*ISM1)* genes. BTBD3 is a member of the transcription factors family and its functions include cytoskeleton dynamics, ion channel modulation, and protein degradation. *DHRS11* and *ISM1* are highly correlated with the expression of some of the other genes that have been identified among the top outcomes of both the case—control and trio—control meta-analysis and are linked to glutamatergic neurotransmission and signaling. Although no significant genome-wide correlations have been found in the whole sample, the findings indicate that *BTBD3, FAIM2*, correlated with *DHRS11*, and adenylate cyclase type 8 gene (*ADCY8)*, correlated with *ISM1*, may be active in OCD pathogenesis. In addition, the top two SNPs with the lowest *p*-values were mapped within *DLGAP1*, a gene homologous to *SAPAP*, involved in the post-synaptic density of glutamatergic synapses.

Another GWAS was conducted by the OCGAS and published by Mattheisen et al. (46). This study is comprised of 1,406, comprehensively assessed, early onset, OCD patients combined with population-based samples. The smallest *p*-value (*p* = 4.13 × 10^−7^) was observed for the locus rs4401971, mapped near protein tyrosine phosphatase receptor type D (*PTPRD)* gene, which is responsible for differentiation of glutamatergic and, together with SLIT and NTRK Like Family Member 3 (*SLITRK3*) gene, GABAergic synapses. The second strongest correlation result was located in the cadherin cluster area. Compared to the hit regions found in the GWAS performed by Stewart et al. (45), 12 of the 15 strongest signals in the sample demonstrated correlations with the same direction of effects (sign test *p* = 0.0176). In regard to the region of Discs Large Homolog Associated Protein 1 (*DLGAP1*) gene, different outcomes were obtained. In the case-control analysis conducted by IOCDF-GC (45), signals in this gene reached top value. Even though in a study by Mattheisen et al. (46) the same significance was not detected, a nearby marker showed a significant value of *p* = 2.67 × 10^−4^, suggesting the association exists. Moreover, the region containing Glutamate Ionotropic Receptor Kainate Type Subunit 2 *(GRIK2)* gene, which rendered as a top signal in IOCDF-GC study, showed nominal, although not experiment-wide (*p* = 0.045), significance in the one performed by OCGAS (46). Finally, in a gene-set study for high-confidence interactions (51), the *DLGAP1* and *GRIK2* revealed a pattern of association and pointed to the possible role of *DLGAP1* and *GRIK2* interactors in the etiology of OCD, including genes such as Neuronal Differentiation 6 (*NEUROD6)* gene, Synaptic Vesicle Glycoprotein 2A gene *(SV2A)*, Glutamate Ionotropic Receptor AMPA Type Subunit 4 gene (*GRIA4)*, and Solute Carrier Family 1 Member 2 gene (*SLC1A2)*. Other associations have been found with IQ Motif Containing K (*IQCK*) gene (*p* < 1 × 10^−6^ with experiment-wide significance) and Orofacial Cleft 1 Candidate 1 (*OFCC1)* (*p* = 6.29 × 10^−5^).

The two above-mentioned studies were meta-analyzed (13), and the results detected an absence of genome-wide significant SNPs: rs4733767 [*p* = 7.1 × 10^−7^; Cancer Susceptibility 8 and Cancer Susceptibility 11 genes (*CASC8/CASC11*)], rs1030757 [*p* = 1.1 × 10^−6^; Glutamate Ionotropic Receptor Delta Type Subunit 2 gene (*GRID2*)], and rs12504244 [*p* = 1.6 × 10^−6^; Proto-Oncogene, Receptor Tyrosine Kinase gene, (*KIT*)] were marked as top haplotypic blocks, while the top signals were localized within or around Ankyrin Repeat And SOCS Box Containing 13 gene (*ASB13)*, R-Spondin 4 gene (*RSPO4)*, Disks large-associated protein 1 gene (*DLGAP1)*, Receptor-type tyrosine-protein phosphatase delta gene (*PTPRD), GRIK2*, Fas Apoptotic Inhibitory Molecule 2 gene (*FAIM2)*, and Cadherin 20 gene (*CDH20)*. Typical heritability variance of OCD was estimated by combined analyses of both samples resulting in a value of 25–30%. The findings of this meta-analysis confirm some of the conclusions of two prior OCD GWASs, with glutamatergic system genes, such as *GRID2, DLGAP1*, being involved in OCD pathogenesis.

Another study examining genetic basis of OCD was performed by den Braber et al. (47). This study included a homogeneous population from the Netherlands. Heritability of OCD, based on SNP analysis, was estimated to be 14% and one SNP, rs8100480, appeared to be significantly associated with OCD in GWAS (*p* = 2.56 × 10^−8^). Additionally, four more genes, Myocyte enhancer binding factor 2B (*MEF2BNB)*, DNA-binding protein RFXANK gene (*RFXANK), MEF2BNB-MEF2B*, and *MEF2B*, were found to be involved in OCD etiology.

Additionally, attempts were made to demonstrate differences in the structure of the CNS in people with OCD compared to the general population. Hibar et al. (63) investigated the relationship between data obtained in GWAS of OCD by Stewart et al. (45) and data of a large-scale meta-analysis by the ENIGMA Consortium (64). Proof of substantial, positive correspondence between variants linked to the greater nucleus accumbens and the putamen volumes and OCD risk variants was identified. Additionally, the putamen, amygdala, and thalamus were brain regions which showed correlation with genetic risk of OCD.

It is worth mentioning that some scientists dealing with the subject of the genetic determinants of OCD have explored sex differences. In the study performed by Khramtsova et al. (51), two genes (*GRID2* and G Protein-Coupled Receptor 135, *GPR135*) were found to be associated with OCD exclusively in females, but there were no genome-wide associations found in either genotype–sex interaction analysis or sex-stratified GWAS. Moreover, heritability of OCD did not differ and there were no significant distinctions in the cross-trait genetic correlations between sexes. The highest variability of effect size between males and females was reached for SNPs linked to gene regulatory function (eQTLs) in the immune system and brain.

## GWAS Findings in OCS

Just recently, Burton et al. (56) examined genetic variants associated with obsessive-compulsive symptoms (OCS) and tested whether OCS and OCD shared genetic risk. The authors carried out GWAS of OCS using the Toronto Obsessive-Compulsive Scale (TOCS) in 5018 unrelated Caucasian children and adolescents. A locus tagged by rs7856850 in an intron of *PTPRD* (protein tyrosine phosphatase δ) was significantly associated with OCS at the genome-wide significance level (*p* = 2.48 × 10^−8^). rs7856850 was also associated with OCD in a meta-analysis of OCD case/control genome-wide datasets (*p* = 0.0069). Obsessive-compulsive symptoms polygenic risk score was correlated with OCD (*p* < 0.01). Obsessive-compulsive symptoms was highly, but not significantly, genetically correlated with OCD (*p* = 0.062).

Smit et al. (55) performed GWAS of obsessions, including ruminations and impulsions, and compulsions, such as checking, washing, and ordering/precision, assessed by subscales of the abbreviated edition of the Padua Inventory. While the obsession subscale and the total Padua score reached insignificant values, the compulsion subscale demonstrated a strong positive genetic association with the case-control OCD GWAS (*p* = 0.017) conducted prior to the analysis by the Psychiatric Genomics Consortium (PGC-OCD). Similar to the studies mentioned above, there were no significant SNPs identified in the study. In addition to the *KIT* and *GRID2* genes, which were previously described, the study showed potential impact of two novel genes, WD repeat-containing protein 7 gene (*WDR7)* and AarF domain-containing protein kinase 1 gene (*ADCK1)*. Genes expressed in the hippocampus, amygdala, and caudate nucleus were correlated with OCS. Moreover, gene-level analyses demonstrated increased correlation with brain regions involved in the reward system, emotions, memory, and fear-formation and enrichment for genes linked to psychiatric conditions.

Alemany-Navarro et al. (53) also tested whether a relationship exists between genes and specific obsessions and/or compulsions. There was no correlation between SNPs and OCD dimensions at the genome-wide level (*p* < 5 × 10^−8^). One gene, SET Domain Containing 3 gene (*SETD3)*, reached genome-wide significant association with hoarding (*p* = 1.89 × 10^−8^), while another, Carboxypeptidase E gene (*CPE)*, was found to be linked to aggressive symptoms (*p* = 4.42 × 10^−6^). Aggressive symptoms were also associated with zinc ion response and lipid metabolism. Among other pathways, ordering OCS were correlated with lipid metabolism, while sexual/religious OCS with G protein-mediated processes; finally, hoarding was correlated with metabolic processes and anion transport. In another study, performed by Bralten et al. (65), genetic correlations between OCD/OCS in the general population and insulin signaling in the central and peripheral nervous system were found. In this study, total OCS score and OCS factors from an exploratory factor analysis were the subject of GWAS in the population-based Philadelphia Neurodevelopmental Cohort (650 children and adolescents). The Spit for Science cohort (5,047 children and adolescents) served to validate the Bralten et al. findings. Researchers used PRS to evaluate shared genetic basis between clinical OCD, the total OCS score, and OCS factors. Gene-set analyses were then conducted with a set of OCD-linked genes focused on central nervous system (CNS) synaptic activity controlled by insulin and analyzed for five peripheral insulin-related traits based on PRS. The authors found a common genetic basis between OCD and “guilty taboo thoughts” and a correlation between CNS, insulin-linked, gene-sets and symmetry/counting/ordering in the Philadelphia Neurodevelopmental Cohort, while the association between “symmetry/counting/ordering” and “contamination/cleaning” found in the Spit for Science cohort was confirmed. Genetically-determined, peripheral, insulin-related, signaling traits such as type 2 diabetes were found to be related to aggressive taboo thinking while genetically-determined, fasting, insulin levels and 2 h glucose levels were correlated with OCD.

## Genomic Relationships With Other Disorders

Researchers have also attempted to answer the question about whether links exist between OCD and other disorders. One of the most widely described associations is the link between tics and OCD. In the study conducted by Yu et al. (66), there were no genome-wide significant SNPs. PRS for OCD was found to be significant (*p* = 2 × 10^−4^), predicting 3.2% of the phenotypic variance in an independent data set, in contrast to non-significant polygenic component in GTS, predicting only 0.6% of the phenotypic variance (*p* = 0.06). Finally, across OCD and GTS there was no significant polygenic signal present. In the study conducted by Davis et al. (67) variance in predisposition to GTS and OCS was assessed and heritability point was evaluated to be 0.58 (*se* = 0.09, *p* = 5.64 × 10^−12^) and 0.37 (*se* = 0.07, *p* = 1.5 × 10^−7^), respectively. Moreover, 21% of the GTS heritability was connected to SNPs with a minor allele frequency of <5%, while in the case of OCD they accounted for 0% of the heritability. Genetic correlation between OCD and GTS reached the value of 0.41 (*p* = 0.002) in this study.

Associations between anorexia nervosa (AN) and OCD have also been analyzed. The aim of the study by Yilmaz et al. (68) was to evaluate the genetic origin of these two disorders, however, no significant genome-wide results for shared AN–OCD risk were found. Despite the absence of significant hits, prominent, reliable signals were located in the leucine-rich repeat-containing 16A gene (*LRRC16A)*, both for AN (*p* = 4.19 × 10^−5^) and OCD (*p* = 1.53 × 10^−3^); upstream of *KIT* gene, both for AN (*p* = 1.62 × 10^−6^) and OCD (*p* = 0.011). In this study, a high genetic association between AN and OCD (*rg* = 0.49 ± 0.13, *p* = 9.07 × 10^−7^) and a sizable SNP heritability (SNP h2 = 0.21 ± 0.02) for the cross-disorder phenotype were reported.

Another disorder suspected to be associated with OCD at the genome level is attention deficit hyperactivity disorder (ADHD). According to the study by Ritter et al. (69), which aims to identify the potential genetic overlap between the two disorders, none of the SNPs were significant at the genome-wide level, implying the lack of evidence for genetic correlation between these two disorders.

Also, as OCD and autism spectrum disorder (ASD) are both heritable disorders of neurodevelopmental origin, Guo et al. (50) assumed that their genetic bases may share some similarities. rs4785741, located in chromosome 16, was the SNP with the top signal in this study (*p* = 6.9 × 10^−7^). In addition, enrichment analyses showed that the following genes: melanocortin 1 receptor *MC1R*, tubulin Beta 3 (*TUBB3)*, dimethylarginine dimethylaminohydrolase 1 (*DDAH1)*, inositol monophosphatase 2 (*IMPA2)*, and parathyroid hormone 2 receptor (*PTH2R)* could theoretically lead to coexistence of ASD and OCD. Additionally, the application of PRS analyses identified a significant, polygenic component of ASD, predicting 0.11% of the phenotypic variance in an independent OCD data collection. With the use of Genome-wide Complex Trait Analysis, global heritability was estimated to be 0.427 (*se* = 0.093) in OCD and 0.174 (*se* = 0.053) in ASD.

Another disorder investigated in the context of its co-existence with OCD is schizophrenia. This subject was investigated by Costas et al. (54). The Dynamin 3 (*DNM3)* gene, involved in the endocytosis of synaptic vesicles, had a significant association at the gene-based test (*p* = 7.9 × 10^−5^) and appears to possibly be involved in OCD pathogenesis. Significant correlation was observed between disease status in OCD sample and the polygenic risk model of schizophrenia data set (PGC-SCZ2), especially when the major histocompatibility complex region was eliminated.

Some investigators tried to examine variety of psychiatric disorders that share pathophysiological background with OCD. Strom et al. (57) examined polygenic heterogeneity across OCD subgroups defined by a comorbid diagnosis. The authors hypothesized that OCD shares common genetic background with other psychiatric comorbidities. In particular, they used a framework of different approaches to study the genetic relationship of OCD with three commonly observed comorbidities, namely major depressive disorder (MDD), attention-deficit hyperactivity disorder (ADHD), and ASD. They found that PRS of such traits as neuroticism, bipolar disorder, AN, age at first birth, educational attainment, and insomnia were significantly associated with OCD across all subgroups. Cross-Disorder Group of the PGC published results of their study investigating genomic relationships, novel loci, and pleiotropic mechanisms across eight psychiatric disorders (52). They performed analyses of 232,964 cases and 494,162 controls from genome-wide studies of AN, ADHD, ASD, bipolar disorder, MDD, OCD, schizophrenia, and TS. As a result they were able to determine three groups of co-related disorders. Meta-analysis across eight disorders revealed 109 loci associated with at least two psychiatric disorders. Detected loci were mainly related to neurodevelopement.

## Treatment Response in OCD

Finally, one study investigated polygenic contributions to therapeutic responses in OCD patients. In the study by Qin et al. (48), which assessed genetic variations potentially influencing sensitivity to selective serotonin reuptake inhibitor (SSRI) treatment, rs17162912, near the Dispatched RND Transporter Family Member 1 (*DISP1)* gene, was the top SNP (*p* = 1.76 × 10^−8^), while rs9303380, rs12437601, rs16988159, rs723815, rs7676822, and rs1911877 were SNPs with possible association. The authors concluded that glutamatergic and serotonergic neurotransmission could be involved in treatment response in OCD. Another GWAS performed by Umehara et al. (49) on the subject of pharmacotherapy in OCD and assessed genetic variants involved in the response to combined SSRI and antipsychotic treatment. Despite the lack of a genome-wide significance level of association between one suggestive SNP and treatment outcomes, five pathways appeared enriched, with the strongest link to calcium signaling pathway.

## Gene-environment Interaction

A number of studies explored the gene-environment interaction in context of OCD. Wang et al. (70) demonstrated interaction between progranulin (*PGRN*) gene and the early trauma on clinical characteristics in patients with OCD. Alemany-Navarro (71) et al. explored the predictive ability of a PRS built from OCD-risk variants, for treatment response in OCD, and the modulation role of stressful life events (SLEs) at the onset of the disorder. The authors failed to demonstrate that PRS predicted treatment response. Nevertheless, PRS predicted basal and post-intervention YBOCS. Importantly, SLEs at onset were not a predictor for treatment response when included in the regression model. Real et al. (72) assessed whether genetic variants in *SLC1A1* and life stress at onset of the disorder interact and modulate pharmacological resistance in OCD. For one SNP (rs3087879), one copy of the risk allele increased the probability of higher treatment resistance. Hemmings et al. (73) investigated interactions between childhood trauma and the BDNF Val66Met variant in patients with OCD. The authors observed no significant association between *BDNF* Val66Met and the development of OCD, but interaction analysis demonstrated that the *BDNF* Met-allele interacted with childhood emotional abuse and increased the risk of OCD.

## Rare Variants in OCD

In recent years, risk gene discovery has also been achieved by studying rare *de novo* (DN) coding variants. For OCD/OCS only two studies have been published so far. Cappi et al. (74) performed whole-exome sequencing in 222 OCD parent-child trios and estimated the contribution of *de novo* mutations to OCD risk and the number of genes involved. The authors identified two high-confidence risk genes, *CHD8* and *SCUBE1*. Just recently, Halvorsen et al. (75) conducted exome sequencing aiming to identify rare damaging coding variants that could influence the occurrence of OCD. In case–control analyses, the most significant result was observed in *SLITRK5* gene. All in all, it could be concluded that there is a contribution of rare variants to OCD, but more replication studies are needed.

## Conclusions: Levering Big Data to Personalize Treatment for OCD?

The emergence of Big Data collaborations in OCD and innovative technologies has afforded new insights into OCD such as discovery of new genetic and pathophysiological pathways involved in this disorder. This stays in line with the genomic studies regarding other neuropsychiatric disorders, such as GTS (35), anxiety disorder (76), depression (77), ASD (78), or schizophrenia (79), which have demonstrated shared genetic background between different symptoms and comorbidities.

Nevertheless, the results of these studies are still limited by diverse populations included in the studies, especially when it comes to genetic ethnicity, diverse sample sizes, and inclusion criteria. At the moment, the majority of studies are limited to genetically white individuals, and we are still lacking studies that are more inclusive regarding other genetic groups, especially minorities. Similarly, phenotype assessment is not homogeneous between all studies. Disease-specific initiatives usually use more elaborate, physician-implemented instruments, such as YBOCS, while phenotype assessment in population-based studies is based on the more general criteria, primarily ICD classification or self-report. Furthermore, population-based studies may not accurately reflect the population-level phenotype due to certain selection bias, such the “heathy volunteer effect” mentioned by Davis et al. (24). A good example is UKB, where the prevalence of self-report OCD is 0.6%, which is well below known population prevalence estimate of OCD (1–3%). Finally, the sample size achieved in population-based studies is limited and, therefore, biobank samples may be better suited as replication samples rather than as discovery. On the other hand, biobanks contain diverse information (clinical, biomarkers, neuroimaging) usually gathered in the longitudinal fashion. Moreover, the methodology of GWASes and data analysis is also not harmonized. All these factors could contribute to the heterogeneity of results obtained in the studies presented in this article.

Considering the evidence presented in the previous sections, it can be concluded that from the point of view of genetics OCD is a highly heterogenous disorder. This is also reflected in the diverse clinical phenotypes as well as complex responses to treatment. Tools aimed toward developing personalized diagnostic and therapeutic approach in OCD are in dire need. The methodological techniques from the field of genomics are poised to unravel the complexity of personalized medicine. They will enable adjustment of diagnosis and treatment in accordance to individual genetic variability of the patient. Finally, the rapid development of bioinformatics and its application to medicine will also render new possibilities. In particular, artificial intelligence and one of its varieties, machine learning, are already used to diagnose (80), predict severity and outcome (81, 82), and trajectories of treatment response (83–85) in OCD. The advancements promised by Big Data catapulted in the field and provided new insights over the past 10 years. As bioinformatics and innovative technologies become ubiquitous in clinical practice, the present the potential (and promise) of personalized medicine. Another future avenue offers creation of international or even intercontinental databases which gather information about more diverse groups, including minorities.

## Author Contributions

NS and AD conceived and designed the study, acquired data, and wrote the original draft of the manuscript. NS, AD, JM, JP, and AL interpreted the data, reviewed and edited the manuscript, and updated the revised version. All authors contributed to the article and approved the submitted version.

## Conflict of Interest

JP has received research support from the National Institute of Mental Health, PCORI, the TLC Foundation for BFRBs, and Pfizer Pharmaceuticals; speaking honoria and travel expenses from the International OCD Foundation and Tourette Association of America; and book royalties from Guilford and Oxford University Press. JM reports receiving research support from the Tourette Association of American (TAA), the American Academy of Neurology (AAN), the Hilda and Preston Davis Foundation, and the American Psychological Foundation. He has served as a consultant to Bracket Global, Syneos Health, and Luminopia, and also received royalties from Elsevier. The remaining authors declare that the research was conducted in the absence of any commercial or financial relationships that could be construed as a potential conflict of interest.

## Publisher's Note

All claims expressed in this article are solely those of the authors and do not necessarily represent those of their affiliated organizations, or those of the publisher, the editors and the reviewers. Any product that may be evaluated in this article, or claim that may be made by its manufacturer, is not guaranteed or endorsed by the publisher.
